# Development of biomarker combinations for postoperative acute kidney injury via Bayesian model selection in a multicenter cohort study

**DOI:** 10.1186/s40364-018-0117-z

**Published:** 2018-01-12

**Authors:** Allison Meisner, Kathleen F. Kerr, Heather Thiessen-Philbrook, Francis Perry Wilson, Amit X. Garg, Michael G. Shlipak, Peter Kavsak, Richard P. Whitlock, Steven G. Coca, Chirag R. Parikh

**Affiliations:** 1 Department of Biostatistics, University of Washington, Box 357232, Seattle, WA 98195 USA; 2 Program of Applied Translational Research, Yale University School of Medicine and VA Medical Center, 60 Temple Street, Suite 6C, New Haven, CT 06510 USA; 3 Veterans Affairs Medical Center, West Haven, CT 06516 USA; 4 Division of Nephrology, Department of Medicine and Department of Epidemiology and Biostatistics, Western University, London, Canada; 5 Institute for Clinical Evaluative Services (ICES) Western, Room ELL-220, Westminster Tower, London Health Sciences Centre, 800 Commissioners Road East, London, ON N6C 6B5 Canada; 6 Kidney Health Research Collaborative, San Francisco VA Medical Center and University of California at San Francisco School of Medicine, 4150 Clement Street, San Francisco, CA 94121 USA; 7 Department of Pathology and Molecular Medicine, McMaster University, Hamilton, ON L8S 4K1 Canada; 8 Department of Surgery, McMaster University, Hamilton, ON Canada; 9 Icahn School of Medicine at Mount Sinai, One Gustave L. Levy Place, Box 1243, New York, NY 10029 USA; 10 Section of Nephrology, Yale University School of Medicine, 60 Temple Street, Suite 6C, New Haven, CT 06510 USA

**Keywords:** Acute kidney injury, Cardiac surgery, Biomarkers combinations, Prognostic

## Abstract

**Background:**

Acute kidney injury (AKI) is a frequent complication of cardiac surgery. We sought prognostic combinations of postoperative biomarkers measured within 6 h of surgery, potentially in combination with cardiopulmonary bypass time (to account for the degree of insult to the kidney). We used data from a large cohort of patients and adapted methods for developing biomarker combinations to account for the multicenter design of the study.

**Methods:**

The primary endpoint was sustained mild AKI, defined as an increase of 50% or more in serum creatinine over preoperative levels lasting at least 2 days during the hospital stay. Severe AKI (secondary endpoint) was defined as a serum creatinine increase of 100% or more or dialysis during hospitalization. Data were from a cohort of 1219 adults undergoing cardiac surgery at 6 medical centers; among these, 117 developed sustained mild AKI and 60 developed severe AKI. We considered cardiopulmonary bypass time and 22 biomarkers as candidate predictors. We adapted Bayesian model averaging methods to develop center-adjusted combinations for sustained mild AKI by (1) maximizing the posterior model probability and (2) retaining predictors with posterior variable probabilities above 0.5. We used resampling-based methods to avoid optimistic bias in evaluating the biomarker combinations.

**Results:**

The maximum posterior model probability combination included plasma N-terminal-pro-B-type natriuretic peptide, plasma heart-type fatty acid binding protein, and change in serum creatinine from before to 0–6 h after surgery; the median probability combination additionally included plasma interleukin-6. The center-adjusted, optimism-corrected AUCs for these combinations were 0.80 (95% CI: 0.78, 0.87) and 0.81 (0.78, 0.87), respectively, for predicting sustained mild AKI, and 0.81 (0.76, 0.90) and 0.83 (0.76, 0.90), respectively, for predicting severe AKI. For these data, the Bayesian model averaging methods yielded combinations with prognostic capacity comparable to that achieved by standard frequentist methods but with more parsimonious models.

**Conclusions:**

Pending external validation, the identified combinations could be used to identify individuals at high risk of AKI immediately after cardiac surgery and could facilitate clinical trials of renoprotective agents.

**Electronic supplementary material:**

The online version of this article 10.1186/s40364-018-0117-z) contains supplementary material, which is available to authorized users.

## Background

Acute kidney injury (AKI) is a frequent complication of cardiac surgery (prevalence: 17–49%) and has serious implications for long-term health [1]. AKI is typically diagnosed on the basis of an increase in serum creatinine over preoperative levels, which often does not occur until several days after the initial injury. Identifying individuals at high risk for AKI immediately following surgery could lead to improved patient outcomes and the development of novel treatment strategies, and is cited as an important step in preventing AKI after cardiac surgery [1].

One strategy for identifying individuals at high risk of AKI after cardiac surgery is to develop a multivariable prognostic model. With that overarching aim in mind, our goal was to identify combinations of variables with strong evidence of prognostic capacity. A first step in developing such combinations is selecting candidate predictors. In the setting of cardiac surgery, most clinical risk factors have modest associations with AKI [2]. One exception is cardiopulmonary bypass (CPB) time, which is strongly associated with AKI after cardiac surgery and has a clear biological relationship with the development of AKI [3, 4]. In addition to CPB time, several biomarkers of kidney injury, inflammation, and cardiac function have been shown to have strong associations with AKI [5–8]. These biomarkers offer the potential to identify patients at high risk of AKI after cardiac surgery, and their application has been encouraged by several consensus conferences [9].

Although CPB time and several biomarkers are strongly associated with risk of AKI, their individual prognostic capacity is modest [5, 6]. However, it may be possible to construct combinations of these variables with higher prognostic capacity. Pursuing such a combination is also biologically motivated since CPB time can be considered to be a measure of the degree of insult to the kidney while the postoperative biomarkers may reflect the response to this insult. Thus, we used Bayesian model averaging (BMA) methods to identify prognostic combinations of postoperative biomarkers (Table 1) and CPB time in a large, multicenter cohort of cardiac surgery patients.

**Table 1:** Candidate biomarkers (measured 0–6 h after surgery)

Category	Biomarker	Abbreviation	Source
Biomarkers of kidney injury	Kidney injury molecule-1 [11]	KIM-1	Urine
	Liver fatty acid-binding protein [11]	L-FABP	Urine
	Cystatin C [12]		Urine
	Albumin [10, 13]		Urine
	Neutrophil gelatinase-associated lipocalin [5]	NGAL	Urine, plasma
	Interleukin-18 [5]	IL-18	Urine
Biomarkers of kidney function	Creatinine [5, 14]	Cr	Urine, serum^a^
Biomarkers of cardiac function	Heart-type fatty acid binding protein [15]	h-FABP	Plasma
	Brain natriuretic peptide [16]	BNP	Plasma
	High-sensitivity troponin T [16]	TNTHS	Plasma
	N-type pro-B-type natriuretic peptide [16]	NT-proBNP	Plasma
	Creatine kinase-MB [16]	CKMB	Plasma
	Troponin I [16]	TNI	Plasma
Biomarkers of inflammation	Interleukin-6 [7]	IL-6	Plasma
	Interleukin-10 [7]	IL-10	Plasma
	Monocyte chemotactic protein-1 [17]	MCP-1	Plasma
	Epidermal growth factor [17]	EGF	Plasma
	Vascular endothelial growth factor [17]	VEGF	Plasma

^a^Also considered the change from preoperative serum creatinine to 0–6 h postoperative and the average of preoperative and 0–6 h postoperative serum creatinine

## Methods

### Study population

This is a secondary analysis of the Translational Research Investigating Biomarker Endpoints in AKI (TRIBE-AKI) study. This study enrolled adults undergoing coronary artery bypass graft (CABG) and/or valve surgery at six academic medical centers in North America between July 2007 and December 2009. Enrollment criteria included increased risk for AKI by any of the following criteria: emergency surgery, preoperative serum creatinine >2 mg/dL, ejection fraction <35% or grade 3 or 4 left ventricular dysfunction, age > 70 years, diabetes mellitus, concomitant CABG and valve surgery, or repeat revascularization surgery. In addition, individuals with evidence of AKI before surgery, prior kidney transplantation, preoperative serum creatinine level > 4.5 mg/dL, or end-stage renal disease were excluded. All participants provided written informed consent and each institution’s research ethics board approved the study.

### Sample collection

Urine and EDTA plasma specimens were collected preoperatively and daily for up to five postoperative days. The first postoperative samples were collected soon after admission to the intensive care unit (0–6 h after surgery). The present investigation considers biomarkers measured at this time point.

Fresh urine samples were obtained from the urimeter of the Foley catheter system and were centrifuged to remove cellular debris. Blood was collected in EDTA tubes and centrifuged to separate plasma. Urine supernatant and plasma were aliquoted into bar-coded cryovials and stored at −80 °C until biomarker measurement. No additives or protease inhibitors were added. Additional details regarding sample collection and storage were provided in earlier reports [5].

### Biomarkers

We included 15 blood and 7 urine biomarkers in this study (Table 1), including three variations of serum creatinine: first postoperative (measured 0–6 h after surgery), absolute difference between preoperative and first postoperative, and average of preoperative and first postoperative. Biomarker measurements were detailed in prior publications [5, 7, 10–17].

### Outcome definitions

The primary outcome was sustained mild AKI, defined as an increase of 50% or more in serum creatinine over preoperative levels lasting at least 2 days during the hospital stay. We chose the sustained mild AKI definition to identify patients most likely to have true kidney injury and to limit misclassification of controls with isolated elevations in serum creatinine due to laboratory variation in creatinine assay, volume disturbances, or hemodynamic derangements [18]. We also considered severe AKI, defined as an increase in serum creatinine of 100% or more or dialysis during hospitalization, as a secondary outcome. Preoperative serum creatinine, collected within 2 months prior to surgery, served as baseline. Pre- and postoperative serum creatinine were measured by the same laboratory for each patient at all centers.

As a secondary analysis, we considered the outcomes of death from all causes at 1 year and 3 years after surgery, which were observed without censoring. We obtained vital status after discharge through various mechanisms. For participants living in the United States, we performed phone calls to patients’ homes, searched the National Death Index, and reviewed hospital records. For Canadian participants, we used phone calls, as well as data held at the Institute for Clinical Evaluative Sciences (ICES) to acquire vital status. The death status and date of death were recorded. These datasets were linked using unique, encoded identifiers and analyzed at ICES.

### Statistical methods

#### Primary analysis

We used BMA methods to identify combinations of biomarkers and CPB time. All biomarkers were log-transformed and CPB time was included as a linear term. Urine biomarkers were not normalized to urine creatinine, though urine creatinine was included as a candidate predictor.

BMA involves assigning each variable a prior probability of being useful for prediction; these prior variable probabilities induce a prior probability for each combination, where the combinations are defined by allowing CPB time and each biomarker to either be included or excluded. The method combines these prior probabilities and the data via Bayes’ theorem to calculate a posterior probability for each combination (“posterior model probability”) and a posterior probability for each variable (“posterior variable probability”) [19–21]. The posterior model probability is a measure of the degree to which the model is supported by the data [22]. Similarly, the posterior variable probability reflects the support in the data for the variable as a predictor of the outcome [23]. The BMA framework can be used for variable selection on the basis of posterior model probabilities or posterior variable probabilities. The BMA approach considers all possible combinations and applies a “leaps and bounds” algorithm to identify the most promising combinations for further consideration; this process provides computational feasibility for searching the large space of candidate models (8,388,608 candidate models given 23 candidate predictors) [19].

In our implementation of BMA, we assigned each biomarker and CPB time a prior probability of ½, meaning that each predictor was a priori as likely to be in the model as not. It is possible to incorporate prior information into these probabilities, but we elected to treat all of the candidate predictors equally. These prior probabilities yield a prior probability for each combination of (0.5)^23^ = 1.19 × 10^−7^, as there are 23 candidate variables. To account for possible center differences, we considered center-adjusted combinations by forcing center to be included in each combination evaluated by BMA. We pre-specified to select two combinations on the basis of the BMA analysis: (1) the maximum posterior model probability combination (the combination with the highest posterior model probability) and (2) the median probability combination (the combination consisting of all predictors with posterior variable probability exceeding 50%) [21].

**Fig. 1:**
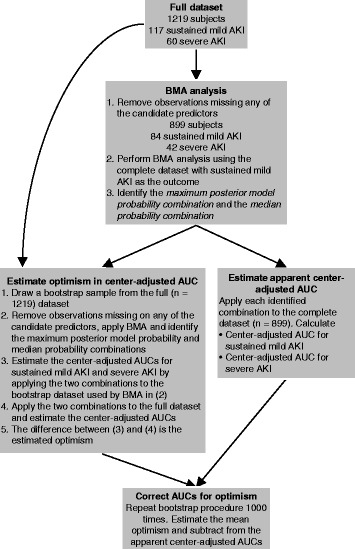
Analysis flow. *Legend:* Abbreviations: AKI = acute kidney injury; BMA = Bayesian model averaging; AUC = area under the receiver operating characteristic curve

We applied BMA to our data to develop combinations for predicting sustained mild AKI. After identifying the maximum posterior model probability combination and the median probability combination, we fit a center-adjusted logistic regression to the biomarkers included in these combinations, with sustained mild AKI as the outcome. Using the estimates from these regressions, we estimated the center-adjusted and optimism-corrected area under the receiver operating characteristic curve (AUC) of each combination for sustained mild AKI and for our secondary outcome, severe AKI. First, we estimated the apparent center-adjusted AUC for each combination and each outcome [24]. Then, we estimated the optimism in the center-adjusted AUC for each combination and each outcome using a bootstrapping procedure with 1000 replications [25]. In each bootstrap sample we repeated the entire model selection process. We subtracted the average optimism across bootstrap datasets from the apparent center-adjusted AUC to estimate the center-adjusted and optimism-corrected AUC. Figure 1 describes the analysis in detail. Importantly, this approach addresses model selection bias, resubstitution bias, and potential bias due to center differences [26]. We emphasize that without optimism correction, estimated AUCs will tend to be overestimated due to both resubstitution bias (i.e., using the same data to develop and evaluate a combination) and model selection bias (i.e., using the data to select the model). By accounting for these sources of optimistic bias, we have a more realistic assessment of how the combinations may perform in independent data. This procedure does not supplant external validation. Rather, this is a form of internal validation where the full dataset is used to fit the combination and estimate its apparent performance, followed by bootstrapping to quantify the optimistic bias in the apparent performance. Confidence intervals (CIs) were estimated for the center-adjusted and optimism-corrected AUC by bootstrapping the BMA procedure and obtaining a 95% CI for the apparent center-adjusted AUC, and then shifting the confidence interval by the average optimism.

Our primary measure of model performance was the AUC, which measures how well a combination discriminates cases from controls. We acknowledge the limitations of the AUC and that it represents an incomplete assessment. Our goal was to propose combinations with high prognostic capacity and the potential to be developed into useful risk prediction models, and we were particularly concerned with avoiding common sources of bias in identifying prognostic combinations, including possible center differences [27]. The adjustment for center does not allow for individual predicted risks. Therefore, we do not assess model calibration in this work, as we do not propose risk prediction models. However, if these combinations are later developed into risk prediction models, an assessment of calibration will be required.

We considered several model diagnostics, including the posterior model probability of the selected combinations across bootstrap samples, the posterior variable probability of each predictor across bootstrap samples, the posterior variable probability of each predictor omitting each observation in turn, and the performance of the estimated selected combinations across bootstrap samples.

#### Exploratory analysis

In an exploratory analysis, we compared the performance of the BMA procedure to two common variable selection methods: forward selection and univariate selection. The following algorithm was used to compare the three methods. We randomly split the data into training and test datasets of equal size with equal numbers of sustained mild AKI cases. We then applied each of the three model selection methods to the training data. First, we applied BMA and identified the maximum posterior model probability combination and the median probability combination. Second, we applied forward selection with a *p*-value threshold of 0.1. Third, we applied univariate selection, forming a combination of all variables with a p-value less than 0.1. All methods used center-adjustment. In each iteration we applied the resulting combinations to the test data and estimated the center-adjusted AUC for the combination using the test data only; thus, we performed internal validation whereby the training dataset was used for fitting while the test dataset was held out for evaluation. We repeated this procedure 1000 times, independently randomly splitting the data into training and test datasets each time. We calculated 95% intervals as the 2.5th and 97.5th percentiles of the AUC across these 1000 replications.

#### Secondary analysis

As a secondary analysis, we evaluated the association of the biomarker combinations identified by the BMA methods with death at 1 year and 3 years after surgery. For each biomarker combination and each time point (1 year and 3 years), we fit a logistic regression model with the fixed estimated biomarker combination, adjusting for center. We used the full dataset to estimate the odds ratio describing the association between the combination and death. We can consider the two estimated combinations, *M*_*1*_ and *M*_*2*_, where *M*_*1*_ has *p* variables (denoted by *X*), combined via the parameters *β*_1_, …, *β*_2_, and *M*_*2*_ has *q* variables (denoted by *Y*), combined via the parameters *α*_1_, …, *α*_*q*_:

$$ {M}_1={\beta}_1{X}_1+\dots +{\beta}_p{X}_p $$

$$ {M}_2={\alpha}_1{Y}_1+\dots +{\alpha}_q{Y}_q. $$

The odds ratio for the association between the combination and death was estimated by fitting two logistic regressions for each time point (1 year and 3 years):

$$ logit\ P\left( Death| Center,{M}_1\right)={\delta}_0^C+{\delta}_1{M}_1 $$

$$ logit\ P\left( Death| Center,{M}_2\right)={\theta}_0^C+{\theta}_1{M}_2, $$

where ${\delta}_0^C$ and ${\theta}_0^C$ are center-specific intercepts.

All analyses were completed using R 3.1.2. The *BMA* package in R was used for the BMA analyses [28]. The R code for the primary analysis is provided in (Additional file 1: Item S1) and at https://github.com/allisonmeisner/BMAbiomarkers.

## Results

Table 2 characterizes the study population. There were 1219 patients in the full dataset, including 117 sustained mild AKI cases and 60 severe AKI cases (55 patients had both outcomes). Approximately 300 individuals were missing one or more candidate variable measurements and were excluded from the BMA analysis, leaving 899 observations, including 84 sustained mild AKI cases and 42 severe AKI cases (Fig. 1). The prevalence of sustained mild AKI and severe AKI were similar among the individuals with and without missing data.

**Table 2:** Demographics and clinical variables by sustained mild AKI status

	Overall (1219)	Sustained Mild AKI	
		Non-event (1102)	Event (117)
Demographics			
Age (years), *mean (SD)*	71.5 (10.1)	71.5 (10.1)	71.1 (10.5)
Male sex*, n (%)*	826 (68%)	749 (68%)	77 (66%)
White race, *n (%)*	1141 (94%)	1034 (94%)	107 (91%)
Center, *n (%)*			
1	109 (9%)	102 (9%)	7 (6%)
2	67 (5%)	57 (5%)	10 (9%)
3	104 (9%)	88 (8%)	16 (14%)
4	534 (44%)	474 (43%)	60 (51%)
5	51 (4%)	43 (4%)	8 (7%)
6	354 (29%)	338 (31%)	16 (14%)
Clinical variables			
Preoperative eGFR (mL/min per 1.73 m^2^), *mean (SD)*	67.2 (19.4)	67.5 (18.9)	64.9 (23.2)
Diabetes, *n (%)*	480 (39%)	427 (39%)	53 (45%)
Hypertension, *n (%)*	961 (79%)	863 (78%)	98 (84%)
Congestive heart failure, *n (%)*	314 (26%)	264 (24%)	50 (43%)
Type of surgery, *n (%)*			
CABG or valve	963 (79%)	883 (80%)	80 (68%)
CABG and valve	255 (21%)	218 (20%)	37 (32%)
Status of procedure, *n (%)*			
Elective	964 (79%)	883 (80%)	81 (69%)
Urgent or emergent	255 (21%)	219 (20%)	36 (31%)
Cardiac catheterization <48 h prior to surgery, *n (%)*	73 (6%)	66 (6%)	7 (6%)
Preoperative myocardial infarction, *n (%)*	313 (26%)	279 (26%)	34 (29%)
Reoperation, *n (%)*	155 (13%)	143 (13%)	12 (10%)
CPB time (minutes), *mean (SD)*	114.2 (59.9)	109.7 (54.1)	155.8 (88.2)
Severe AKI, *n (%)*	60 (5%)	5 (<1%)	55 (47%)
Biomarkers			
Postoperative serum creatinine (mg/dL), *median (IQR)*	1.0 (0.8, 1.3)	1.0 (0.8, 1.2)	1.3 (1.1, 1.7)
Change in serum creatinine (mg/dL), *median (IQR)*	0 (−0.10, 0.11)	0 (−0.14, 0.10)	0.20 (0, 0.38)
Average serum creatinine (mg/dL), *median (IQR)*	1.1 (0.9, 1.2)	1.0 (0.9, 1.2)	1.2 (1.0, 1.5)
Postoperative urine markers, *median (IQR)*			
Creatinine (mg/dL)	23.7 (12.0, 41.3)	22.9 (11.6, 40.6)	31.4 (17.1, 49.7)
IL-18 (pg/mL)	11.6 (4.1, 42.3)	10.6 (3.9, 35.0)	35.3 (10.6, 235.2)
NGAL (ng/mL)	10.2 (4.1, 51.5)	9.5 (3.9, 42.1)	26.0 (6.8, 178.5)
Albumin (mg/L)	14.6 (6.9, 39.5)	13.6 (6.6, 36.1)	25.1 (12.6, 68.0)
KIM-1 (ng/mL)	0.44 (0.17, 0.99)	0.40 (0.16, 0.90)	0.96 (0.47, 1.78)
L-FABP (ng/mL)	19.1 (4.3, 105.2)	17.6 (4.0, 98.4)	52.0 (7.4, 397.9)
Cystatin C (mg/L)	0.17 (0.05, 0.26)	0.16 (0.05, 0.26)	0.21 (0.10, 0.30)
Postoperative plasma markers, *median (IQR)*			
BNP (pg/mL)	53.4 (25.9, 130.1)	50.6 (24.8, 117.6)	111.7 (49.2, 248.7)
NGAL (ng/mL)	185.6 (118.6, 268.2)	178.3 (114.8, 258.9)	244.4 (180.9, 338.8)
IL-10 (pg/mL)	44.7 (13.6, 109.9)	42.9 (13.1, 109.5)	56.5 (25.5, 110.9)
IL-6 (pg/mL)	165.5 (91.4, 295.6)	155.9 (87.8, 276.5)	338.3 (161.0, 576.3)
NT-proBNP (pmol/L)	57.1 (22.5, 142.2)	48.3 (21.5, 123.8)	138.0 (66.9, 291.6)
TNI (μg/L)	1.5 (0.8, 3.2)	1.5 (0.8, 2.9)	2.9 (1.5, 6.9)
TNTHS (ng/L)	406.3 (249.4, 757.0)	392.9 (243.6, 695.6)	744.2 (356.4, 1602.5)
CKMB (μg/L)	21.8 (14.1, 37.3)	21.5 (13.7, 34.5)	31.6 (18.6, 60.8)
h-FABP (μg/L)	31.2 (21.1, 49.4)	30.0 (20.7, 46.1)	55.4 (34.7, 141.0)
MCP-1 (pg/mL)	449.8 (306.9, 734.6)	434.5 (302.2, 713.8)	518.8 (384.7, 883.0)
EGF (pg/mL)	0.90 (0.90, 3.55)	0.90 (0.90, 3.69)	0.90 (0.90, 0.90)
VEGF (pg/mL)	4.5 (4.5, 4.5)	4.5 (4.5, 4.5)	4.5 (4.5, 4.5)

Abbreviations: *AKI* acute kidney injury, *SD* standard deviation, *IQR* interquartile range, *eGFR* estimated glomerular filtration rate, *CABG* coronary artery bypass graft, *CPB* cardiopulmonary bypass, *IL-18* interleukin-18, *NGAL* neutrophil gelatinase-associated lipocalin, *KIM-1* kidney injury molecule-1, *L-FABP* liver fatty acid-binding protein, *BNP* brain natriuretic peptide, *IL-10* interleukin-10, *IL-6* interleukin-6, *NT-proBNP* N-terminal-pro-B-type natriuretic peptide, *TNI* troponin I, *TNTHS* high-sensitivity troponin T, *CKMB* creatine kinase-MB, *h-FABP* heart-type fatty acid binding protein, *MCP-1* monocyte chemoattractant protein-1, *EGF* epidermal growth factor, *VEGF* vascular endothelial growth factor

### Primary analysis

Table 3 gives the results from the primary BMA analyses. The maximum posterior model probability combination included plasma N-terminal-pro-B-type natriuretic peptide (NT-proBNP), plasma heart-type fatty acid binding protein (h-FABP), and absolute change in serum creatinine from before to 0–6 h after surgery. The center-adjusted, optimism-corrected AUC for this combination was 0.80 (95% CI: 0.78, 0.87) for sustained mild AKI and 0.81 (0.76, 0.90) for severe AKI. The median probability combination model included plasma interleukin-6 (IL-6), plasma NT-proBNP, plasma h-FABP, and change in serum creatinine. The center-adjusted, optimism-corrected AUC for this combination was 0.81 (0.78, 0.87) for sustained mild AKI and 0.83 (0.76, 0.90) for severe AKI. Recall that these AUCs are estimated by first using the full dataset to fit the combinations and estimate their apparent performance, then applying the bootstrap to estimate the optimistic bias in this apparent performance. For comparison, the biomarker with the highest individual center-adjusted AUC for sustained mild AKI was change in serum creatinine; the center-adjusted AUC for this biomarker alone was 0.76, outside of the 95% CI for the two BMA combinations. The posterior model probability (a measure on the probability scale of the support for the model in the data) for the two combinations was 0.20. (Additional file 1: Figures S1 and S2) illustrate the distribution of the biomarker combinations. (Additional file 1: Figure S3) includes the distributions of three biomarkers among sustained mild AKI controls, stratified by center. These distributions vary by center, providing evidence that center should be taken into account when interpreting the biomarkers. The posterior variable probabilities for each candidate predictors are given in Additional file 1: Table S1.

**Table 3:** Combinations selected by BMA methods and their estimated performance

	Maximum posterior model probability combination	Median probability combination
AUC (95% CI)		
Sustained mild AKI	0.80 (0.78, 0.87)	0.81 (0.78, 0.87)
Severe AKI	0.81 (0.76, 0.90)	0.83 (0.76, 0.90)
Posterior model probability	0.20	0.20
Odds ratios (95% CI)^a^		
Log plasma IL-6^b^		1.58 (1.12, 2.26)
Log plasma NT-proBNP	1.60 (1.28, 2.02)	1.58 (1.26, 1.99)
Log plasma h-FABP	2.00 (1.33, 3.02)	1.85 (1.22, 2.82)
Change in serum Cr^c^	1.80 (1.55, 2.11)	1.79 (1.54, 2.10)
Posterior variable probability		
Log plasma IL-6^b^		0.57
Log plasma NT-proBNP	1.00	1.00
Log plasma h-FABP	0.76	0.76
Change in serum Cr	1.00	1.00

Abbreviations: *BMA* Bayesian model averaging, *AUC* area under the receiver operating characteristic curve, *CI* confidence interval, *AKI* acute kidney injury, *IL-6* interleukin-6, *NT-proBNP* N-terminal-pro-B-type natriuretic peptide, *h-FABP* heart-type fatty acid binding protein, *Cr* creatinine

^a^The odds ratios and corresponding 95% CIs are based on logistic regression with sustained mild AKI as the outcome

^b^The results for plasma IL-6 are given only for the median probability combination as plasma IL-6 was not included in the maximum posterior model probability combination

^c^per 0.1 mg/dL

The model diagnostics considered for BMA (Additional file 1: Figures S4-S7) indicated variability in the posterior model probabilities and posterior variable probabilities across bootstrap samples, as well as some potentially influential observations. Importantly, however, the AUCs of the estimated selected combinations were reasonably stable across bootstrap samples. Finally, in order to explore the impact of deleting observations with missing data, we compared the results of a multiple imputation analysis to the results of our complete-case analysis (Additional file 1: Item S2). We found similar results in terms of the combinations selected and the performance of the selected combinations.

### Exploratory analysis

**Fig. 2:**
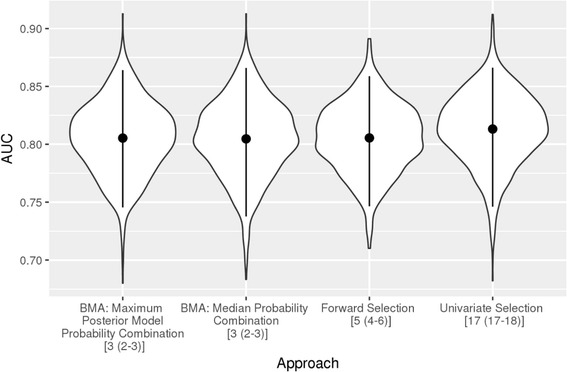
Distribution of AUC estimates by selection approach. *Legend:* Abbreviations: BMA = Bayesian model averaging; AUC = area under the receiver operating characteristic curve.The mean AUC (point) and 2.5th and 97.5th quantiles of AUC (line) across 1000 sample splits are given. The median (interquartile range) combination size for each approach is given in square brackets in the horizontal axis labels

Figure 2 summarizes the results of the analysis comparing BMA, forward selection and univariate selection. For all three methods, incomplete observations were removed, leaving 899 observations. Univariate selection had the highest average AUC by a small margin, although all methods performed comparably. The mean center-adjusted AUC across sample splits (where the combinations were fitted in the training dataset and evaluated in the held out test dataset) was 0.81 (95% interval: 0.75, 0.86), 0.80 (0.74, 0.87), 0.81 (0.75, 0.86) and 0.81 (0.75, 0.87) for the BMA maximum posterior model probability combination, the BMA median probability combination, forward selection, and univariate selection, respectively. The advantage of BMA in these data appears to be parsimony; the median number of predictors included in the selected combinations was three for both BMA combinations, five for forward selection, and 17 for univariate selection.

### Secondary analysis

For the secondary analysis of mortality, individuals missing any of the biomarkers in the selected combinations were excluded, leaving 934 participants. At 1 year after surgery, 41 individuals had died; by 3 years, 89 participants had died. For the outcome of death at 1 year, the maximum posterior model probability combination (plasma NT-proBNP, plasma h-FABP, and change in serum creatinine) had a center-adjusted odds ratio per standard deviation of 1.61 (95% CI: 1.21, 2.15) while the median probability combination (plasma IL-6, plasma NT-proBNP, plasma h-FABP, and change in serum creatinine) had a center-adjusted odds ratio per standard deviation of 1.72 (1.28, 2.31). For death at 3 years, the maximum posterior model probability combination had a center-adjusted odds ratio per standard deviation of 1.61 (1.29, 1.99) while the median probability combination had a center-adjusted odds ratio per standard deviation of 1.72 (1.37, 2.15).

## Discussion

We used BMA methods to develop two biomarker combinations with the potential to identify individuals at high risk of AKI after cardiac surgery. The combinations demonstrated good discriminatory performance as measured by the AUC, even after addressing several sources of bias common in the evaluation of risk prediction models [26]. Furthermore, the combinations performed well not only in identifying individuals at high risk of sustained mild AKI, for which they were constructed, but also the more commonly used outcome of severe AKI. Using the outcome of sustained mild AKI provided a larger sample size than severe AKI, and we believe it limited the number of controls misclassified as cases compared to transient mild AKI. We also provided evidence that the combinations developed to predict sustained mild AKI are associated with mortality. Prior to their adoption, these combinations must be validated externally.

The three novel biomarkers included in the combinations identified in our analysis were plasma NT-proBNP, plasma IL-6, and plasma h-FABP, all of which were positively associated with sustained mild AKI in our data. Plasma NT-proBNP has been previously shown to be positively associated with mortality and cardiovascular disease in patients with stable coronary heart disease [29], with AKI in critically ill patients [30], and with AKI and AKI-associated mortality in patients with acute heart failure [31]. Likewise, plasma IL-6 has been shown to be positively associated with mortality in acute heart failure [32] and with AKI in patients with sepsis [33]. Plasma h-FABP has previously been shown to be positively associated with AKI in patients undergoing cardiac surgery [34].

A limitation of this study is that we developed prognostic biomarker combinations, not risk prediction models. These combinations (if validated) can be used to identify high-risk participants, but, in their current form, they cannot be used to estimate risk of AKI. This is a consequence of accounting for center in our analysis in order to avoid possible bias resulting from differences among centers. Several important steps are required to develop either of the proposed combinations into a risk prediction model: (1) validation of the prognostic capacity of the combination on independent data; (2) standardization of biomarker measurements across centers and laboratories; and (3) transformation of the “combination score” to the risk scale and establishing risk model calibration. Thus, the identification of prognostic combinations represents an intermediate step on the path to a risk prediction model.

This study had several strengths, including its sample size, the number of biomarkers measured, and the use of rigorous statistical methods to assess performance. All statistical analyses were pre-specified, including pre-specification of the summaries to be reported. Our analyses indicate that in these data, the BMA methods yielded combinations with prognostic capacity comparable to that achieved by forward and univariate selection but with smaller models. In other words, the BMA methods offered combinations with similar performance at reduced cost. Such parsimony may be desirable as using a smaller combination may be more affordable and practical. This was achieved without sacrificing computational efficiency: it took 3.2 s to apply BMA to our data using a personal Windows laptop. In addition, there is evidence in our data that the combinations identified by the BMA methods are associated with postoperative mortality. Further research is needed to determine whether these biomarker combinations can be used to identify individuals at high risk of death following cardiac surgery.

If these combinations are found to perform well in independent data, they could be used to enrich clinical trial enrollment, thereby increasing the likelihood of identifying new AKI therapies. For illustrative purposes, we present examples of this strategy, termed “prognostic enrichment” [35], in Table 4. For instance, if a researcher were interested in developing a treatment for severe AKI, he could use the biomarker combinations developed here to calculate a biomarker “score” for prospective trial participants (using each individual’s biomarker values and the estimated coefficients for each biomarker) and enroll individuals above some threshold. If the 75th percentile of the median probability combination was used as a threshold, the sample size required to achieve 90% power (alpha = 0.05) for a treatment that decreases AKI risk by 30% could be reduced from nearly 8200 to 2286 (note that such a strategy would require screening four individuals to identify one eligible for the study).

**Table 4:** Clinical trial enrichment for severe AKI

Threshold	Number to screen to identify 1 eligible patient	Combination/Marker	Severe AKI rate among screen positives (untreated)	Total sample size required
(None)	1	(None)	4.7%	8156
25th percentile	1.3	Maximum posterior probability combination	6.0%	6328
		Median probability combination	6.1%	6166
50th percentile	2	Maximum posterior probability combination	8.0%	4600
		Median probability combination	8.0%	4600
75th percentile	4	Maximum posterior probability combination	14.2%	2450
		Median probability combination	15.1%	2286

Abbreviations: *AKI* = acute kidney injury. We calculated sample sizes for a renopreventive treatment for 90% power, alpha = 0.05, and 30% reduction in AKI risk under treatment. A trial that enrolled only “high risk” patients would require fewer patients in the trial due to a higher event rate, while requiring that more patients be screened in order to identify eligible patients

## Conclusions

Using BMA methods with data from a large, multicenter study, we have developed biomarker combinations and provided strong evidence that they are able to identify patients at high risk of AKI after cardiac surgery in our data. These combinations could be used in the development of treatments for AKI, potentially reducing the associated morbidity and mortality and improving long-term health after cardiac surgery.

## Additional files

Additional file 1: Item S1. R code for the primary analysis. **Figure S1.** Distribution of biomarker combinations in the largest center, stratified by sustained mild AKI case status (scaled). **Figure S2.** Distribution of biomarker combinations in the largest center, stratified by sustained mild AKI case status. **Figure S3.** Distribution of three biomarkers (log plasma NT-proBNP, change in sCr, and log plasma h-FABP) among controls (individuals without sustained mild AKI), stratified by center. **Table S1.** Posterior variable probabilities for each candidate predictor. **Figure S4.** Posterior model probability of the combinations selected by the BMA methods across the 1000 bootstrap samples. The first plot corresponds to the maximum posterior probability combination and the second plot corresponds to the median probability combination. “Truncated” means the combination was not considered by the BMA algorithm in that particular bootstrap sample; the truncated value is the minimum posterior model probability in that sample. “Index” indicates the bootstrap sample number. **Figure S5.** Posterior variable probabilities for each of the candidate predictors across 1000 bootstrap samples. “Index” indicates the bootstrap sample number. **Figure S6.** Posterior variable probabilities for each of the candidate predictors when each patient was left out in turn (only observations non-missing on all candidate predictors were included). “Index” indicates the (arbitrary) rank order of the patient in the analysis dataset. **Figure S7.** Performance (in terms of the center-adjusted AUC) of the estimated selected combinations across 1000 bootstrap samples. The first plot corresponds to the AUC for the outcome of sustained mild AKI; the second plot corresponds to the AUC for the outcome of severe AKI. “Index” indicates the bootstrap sample number. **Item S2.** Multiple imputation analysis. (PDF 2793 kb)

## Abbreviations

AKI: Acute kidney injury
AUC: Area under the receiver operating characteristic curve
BMA: Bayesian model averaging
CABG: Coronary artery bypass graft
CI: Confidence interval
CPB: Cardiopulmonary bypass
h-FABP: Heart-type fatty acid binding protein
ICES: Institute for Clinical Evaluative Sciences
IL-6: Interleukin-6
NT-proBNP: N-terminal-pro-B-type natriuretic peptide
TRIBE-AKI: Translational Research Investigating Biomarker Endpoints in Acute Kidney Injury
