# Supplementary material for: Development of biomarker combinations for postoperative acute kidney injury via Bayesian model selection in a multicenter cohort study
Source: Biomark Res. 2018 Jan 12;6:3. doi: 10.1186/s40364-018-0117-z (PMC5767010; doi:10.1186/s40364-018-0117-z)
Supplement: Supplementary file 1 — R code for the primary analysis. Figure S1. Distribution of biomarker combinations in the largest center, stratified by sustained mild AKI case status (scaled). Figure S2. Distribution of biomarker combinations in the largest center, stratified by sustained mild AKI case status. Figure S3. Distribution of three biomarkers (log plasma NT-proBNP, change in sCr, and log plasma h-FABP) among controls (individuals without sustained mild AKI), stratified by center. Table S1. Posterior variable probabilities for each candidate predictor. Figure S4. Posterior model probability of the combinations selected by the BMA methods across the 1000 bootstrap samples. The first plot corresponds to the maximum posterior probability combination and the second plot corresponds to the median probability combination. “Truncated” means the combination was not considered by the BMA algorithm in that particular bootstrap sample; the truncated value is the minimum posterior model probability in that sample. “Index” indicates the bootstrap sample number. Figure S5. Posterior variable probabilities for each of the candidate predictors across 1000 bootstrap samples. “Index” indicates the bootstrap sample number. Figure S6. Posterior variable probabilities for each of the candidate predictors when each patient was left out in turn (only observations non-missing on all candidate predictors were included). “Index” indicates the (arbitrary) rank order of the patient in the analysis dataset. Figure S7. Performance (in terms of the center-adjusted AUC) of the estimated selected combinations across 1000 bootstrap samples. The first plot corresponds to the AUC for the outcome of sustained mild AKI; the second plot corresponds to the AUC for the outcome of severe AKI. “Index” indicates the bootstrap sample number. Item S2. Multiple imputation analysis. (PDF 2793 kb) [file 40364_2018_117_MOESM1_ESM.pdf]

Item S1: R code for the primary analysis.

Note: This code was tailored the code for the data at hand. The user of this code should check each line to ensure that it is appropriate for his/her data. In addition, the user should be aware that aspects of his/her data may complicate the analysis and/or require modifications to sections of the code.

This code is also provided at https://github.com/allisonmeisner/BMAbiomarkers.

Section I: Setting up the data

```
####
## TRIBE Analysis
## Identifying Combinations of Markers
## Markers: 1st post-op, log transformed
## Clinical covariates: cpb time (continuous)
## Adjust for center
## Adults only
## Outcome: sustained (2+ days) mild AKI
## Method: BMA, forcing center in. Select top model. Calculate center-adjusted AUC.
##    Bootstrap 1000 times. Repeat modeling in each sample, apply to original dataset.
##    Estimate optimism. Subtract from apparent AUC.
####

### Read in data
setwd("…/Data")
dat<-read.csv("TribeDatasetaug272014.csv")
str(dat) ## check out the data
summary(dat)

### Create dataset with our variables (keep adult observations and all (potentially) necessary variables)
datsub<-dat[which(dat$adult==1),c("sercre106","bldbnp106","bl1tngal106","urcre106",
                "ur1il18106","ur1ngal106","urmalb106","uralbcre106","urkim1106","urlfabp106",
                "urcysc106","cur1il18106","cur1ngal106","curkim1106","curlfabp106",
                "curcysc106","bldIL10106","bldIL6106","bldPROBNP106","bldTNI106","bldTNTHS106",
                "bldCKMB106","bldFABP106","bldMCP1106","bldEGF106","bldVEGF106",
                "sercrepre","center","cpbtime","aki2d","aki1d","ndayaki1d")]
summary(datsub)
apply(datsub,2,min,na.rm=T)
### zeroes: urmalb106, uralbcre106, urcysc106, curcysc106 --> these are not errors!
### will need to add a little bit to the 0's to take logs

### Create variables needed
# (1) add a little bit to biomarkers with 0's – these 0's are not errors and we need positive biomarker
values to take logs
# "fix" = add a little bit (half of the minimum of positive values) to the 0's
datsub$urmalb106<-ifelse(datsub$urmalb106==0,min(datsub$urmalb106[which(datsub$urmalb106>0)],
                        na.rm=T)/2,datsub$urmalb106)
datsub$uralbcre106<-ifelse(datsub$uralbcre106==0,
                min(datsub$uralbcre106[which(datsub$uralbcre106>0)],na.rm=T)/2,datsub$uralbcre106)
datsub$urcysc106<-ifelse(datsub$urcysc106==0,min(datsub$urcysc106[which(datsub$urcysc106>0)],
                na.rm=T)/2,datsub$urcysc106)
datsub$curcysc106<-ifelse(datsub$curcysc106==0,min(datsub$curcysc106[which(datsub$curcysc106>0)],
                na.rm=T)/2,datsub$curcysc106)

# (2) take logs of the biomarkers
datsub$Lsercre106<-log(datsub$sercre106)
datsub$Lbldbnp106<-log(datsub$bldbnp106)
datsub$Lbl1tngal106<-log(datsub$bl1tngal106)
datsub$Lurcre106<-log(datsub$urcre106)
datsub$Lur1il18106<-log(datsub$ur1il18106)
datsub$Lur1ngal106<-log(datsub$ur1ngal106)
datsub$Lurmalb106<-log(datsub$urmalb106)
datsub$Luralbcre106<-log(datsub$uralbcre106)
datsub$Lurkim1106<-log(datsub$urkim1106)
```

```
datsub$Lurlfabp106<-log(datsub$urlfabp106)
datsub$Lurcysc106<-log(datsub$urcysc106)
datsub$Lcur1il18106<-log(datsub$cur1il18106)
datsub$Lcur1ngal106<-log(datsub$cur1ngal106)
datsub$Lcurkim1106<-log(datsub$curkim1106)
datsub$Lcurlfabp106<-log(datsub$curlfabp106)
datsub$Lcurcysc106<-log(datsub$curcysc106)
datsub$LbldIL10106<-log(datsub$bldIL10106)
datsub$LbldIL6106<-log(datsub$bldIL6106)
datsub$LbldPROBNP106<-log(datsub$bldPROBNP106)
datsub$LbldTNI106<-log(datsub$bldTNI106)
datsub$LbldTNTHS106<-log(datsub$bldTNTHS106)
datsub$LbldCKMB106<-log(datsub$bldCKMB106)
datsub$LbldFABP106<-log(datsub$bldFABP106)
datsub$LbldMCP1106<-log(datsub$bldMCP1106)
datsub$LbldEGF106<-log(datsub$bldEGF106)
datsub$LbldVEGF106<-log(datsub$bldVEGF106)

# create scr variables – change from pre to post, and average over pre to post
datsub$delta_scr<-datsub$sercre106-datsub$sercrepre
datsub$avg_scr<-0.5*(datsub$sercre106+datsub$sercrepre)

# create outcome – at least 2 days of mild AKI
datsub$susmild<-as.numeric(datsub$ndayaki1d>=2)

# change center to a factor variable – will use later
datsub$centerF<-factor(datsub$center)

# create new cpbtime variable where 0's are assigned to missing values
datsub$cpbtime0<-ifelse(datsub$cpbtime==0,NA,datsub$cpbtime)

### Create final dataset (save only the necessary variables)
datfinal<-datsub[,c("centerF","cpbtime","cpbtime0","Lsercre106","Lbldbnp106","Lbl1tngal106",
                         "Lurcre106","Lur1il18106","Lur1ngal106","Lurmalb106","Luralbcre106",
                         "Lurkim1106","Lurlfabp106","Lurcysc106","Lcur1il18106","Lcur1ngal106",
                         "Lcurkim1106","Lcurlfabp106","Lcurcysc106","LbldIL10106","LbldIL6106",
                         "LbldPROBNP106","LbldTNI106","LbldTNTHS106","LbldCKMB106","LbldFABP106",
                         "LbldMCP1106","LbldEGF106","LbldVEGF106","delta_scr","avg_scr","susmild",
                         "aki2d","aki1d")]
str(datfinal) # check things look OK
write.csv(datfinal,"NewMarkerAnalysis20141216.csv",row.names=FALSE)
```

Section II: Running BMA analyses, including (i) running BMA, (ii) estimating apparent center-adjusted AUC, (iii) estimating optimism in center-adjusted AUC, (iv) estimating 95% CIs for apparent center-adjusted AUC and (v) correcting apparent center-adjusted AUC and 95% CIs for optimism.

```
######################
## Post-operative Biomarker Combinations with BMA
## Proposal: To use BMA to develop combinations of biomarkers at 0-6h post-op to predict AKI
## Primary Outcome: Sustained mild AKI
## Secondary Outcomes: Severe AKI
## Center adjustment
##  - force center in
##  - choose the two best models:
##         (1) the combination with the highest posterior probability; and
##         (2) the median probability combinations (the model that includes all markers with posterior
probability > 0.5)
##  - calculate center-adjusted ROC for the resulting combos
## Incorporate model selection into a bootstrap + estimate optimism
## Biomarkers (each given prior prob = 1/2): log-transformed uncorrected markers, delta scr, avg scr
## Clinical covariates: CPB time (not excluding people w/cpbtime=0)
## Use the same dataset as created for the last analysis
##
## Also apply to severe AKI (incorporate into bootstrap otherwise there would be some optimism)
######################
```

```
library(BMA) # package for running BMA
library(rms) # package for estimating AUC

### Read in data
setwd("…")
datfinal<-read.csv("NewMarkerAnalysis20141216.csv")
datfinal$centerFAC<-factor(datfinal$centerF) ## just making sure it's a factor variable (it already should
be)

### Run BMA on the candidate markers with susmild as the outcome
BMAunc<-bic.glm(datfinal[,c("centerFAC","cpbtime","Lsercre106","Lbldbnp106","Lbl1tngal106","Lurcre106",
                                  "Lur1il18106","Lur1ngal106","Lurmalb106","Lurkim1106","Lurlfabp106",
                                "Lurcysc106","LbldIL10106","LbldIL6106","LbldPROBNP106","LbldTNI106",
                                "LbldTNTHS106","LbldCKMB106","LbldFABP106","LbldMCP1106","LbldEGF106",
                                "LbldVEGF106","delta_scr","avg_scr")],datfinal$susmild,
                                  glm.family="binomial",occam.window=T,prior.param=c(1,rep(0.5,23)))
           ### prior.param: one entry per predictor (even for factors like center)
           ### gives the prior probability for each predictor
           ### here, prior prob for center = 1 (force center into each combination)
           ###       prior prob for other predictors = 0.5 (default)
summary(BMAunc,conditional=T,digits=5) # look at results of BMA

## Choose the model with the highest posterior probability
BMAuncM1<-BMAunc$mle[1,]
           ## the coefficient estimates for the combination with the highest posterior probability
BMA_M1vars<-BMAuncM1[BMAuncM1 != 0]
           ## includes estimates of 0 for predictors not in the combination – remove those
tormM1<-pmatch(names(BMA_M1vars),c("(Intercept)","centerFAC."),dup=TRUE,nomatch=0)
BMA_M1varsNOC<-BMA_M1vars[tormM1==0]
           ## previous 2 lines: remove the intercept and center variables from the combination – isolate
           ## the biomarker combination

## Choose the median prob combination – biomarkers with posterior probability > 50%
BMAuncM2<-BMAunc$probne0 ## posterior probabilities for each candidate predictor
BMA_M2vars<-BMAuncM2[BMAuncM2 > 50] ## choose biomarkers with posterior prob > 50%
tormM2<-pmatch(names(BMA_M2vars),"centerFAC",dup=TRUE,nomatch=0)
BMA_M2varsNOC<-BMA_M2vars[tormM2==0]
           ## previous 2 lines: remove center – isolate the biomarker combination

## Refit the models with the predictors in each combo (w/center)
## Use complete case dataset (used by BMA above) – remove individuals missing any of the candidate
predictors
datfinalCC<-datfinal[complete.cases(datfinal[,c("centerFAC","cpbtime","Lsercre106","Lbldbnp106",
                      "Lbl1tngal106","Lurcre106","Lur1il18106","Lur1ngal106","Lurmalb106",
                      "Lurkim1106","Lurlfabp106","Lurcysc106","LbldIL10106","LbldIL6106","LbldPROBNP106",
                      "LbldTNI106","LbldTNTHS106","LbldCKMB106","LbldFABP106","LbldMCP1106",
                      "LbldEGF106","LbldVEGF106","delta_scr","avg_scr")]),]

## Use logistic regression to fit the combinations identified by BMA to estimate the center-adjusted
## biomarker combination
## use the complete case dataset (datfinalCC)
## the estimated combination returned by BMA_M1glm should be equal to BMAuncM1 since BMAuncM1 includes the
## MLEs
BMA_M1glm<-glm(as.formula(paste("susmild",'~',paste(names(BMA_M1varsNOC),collapse=" + "),'+
                      factor(centerFAC)')),data=datfinalCC,family="binomial", x=TRUE,y=TRUE)
BMA_M1glm
BMA_M2glm<-glm(as.formula(paste("susmild",'~',paste(names(BMA_M2varsNOC),collapse=" + "),'+
                      factor(centerFAC)')),data=datfinalCC,family="binomial", x=TRUE,y=TRUE)
BMA_M2glm

## Get apparent center-adjusted AUC for each model

## maximum posterior probability combination
betaM1<-BMA_M1glm$coefficients[2:(1+length(BMA_M1varsNOC))] ## relevant estimates from the glms above
xbM1<-as.matrix(datfinalCC[,names(betaM1)]) %*% betaM1
                      ## linear predictor based on the estimated combination and the data in the
                      ## complete-case dataset
resultsmatM1<-as.data.frame(cbind(xbM1,datfinalCC$susmild,datfinalCC$centerFAC))
```

```
                      ## create new dataset with the linear predictor, outcome and center
allsomersM1<-sapply(split(resultsmatM1, resultsmatM1[,3]),function(x) somers2(x[,1],x[,2]))[1,]
                      ## split the data by center and estimate the apparent AUC for the linear predictor
                      ## in each center
neventsM1<-sapply(split(resultsmatM1, resultsmatM1[,3]),function(x) sum(x[,2],na.rm=T))
                      ## split the data by center and determine the number of cases/events in each center
aucM1<-(allsomersM1[which(is.na(allsomersM1)==FALSE)] %*% neventsM1[neventsM1>0])/sum(neventsM1,na.rm=T)
                      ## estimate the apparent center-adjusted AUC (weighted average of center-specific
                      ## AUCs, weighted by the number of cases

## median probability combination – same idea as above to get apparent center-adjusted AUC of the
combination
betaM2<-BMA_M2glm$coefficients[2:(1+length(BMA_M2varsNOC))]
xbM2<-as.matrix(datfinalCC[,names(betaM2)]) %*% betaM2
resultsmatM2<-as.data.frame(cbind(xbM2,datfinalCC$susmild,datfinalCC$centerFAC))
allsomersM2<-sapply(split(resultsmatM2, resultsmatM2[,3]),function(x) somers2(x[,1],x[,2]))[1,]
neventsM2<-sapply(split(resultsmatM2, resultsmatM2[,3]),function(x) sum(x[,2],na.rm=T))
aucM2<-(allsomersM2[which(is.na(allsomersM2)==FALSE)] %*% neventsM2[neventsM2>0])/sum(neventsM2,na.rm=T)

##########
## Write a function to estimate CI for apparent center-adjusted AUC and optimism in the apparent center-
## adjusted AUC by bootstrapping

bootBMA<-function(x){
           # 1. bootstrap sample
           bootdat<-datfinal[sample(1:nrow(datfinal),size=nrow(datfinal),replace=TRUE),]
           # 2. do BMA in bootstrap sample
           BMAmod<-bic.glm(bootdat[,c("centerFAC","cpbtime","Lsercre106","Lbldbnp106","Lbl1tngal106",
                      "Lurcre106","Lur1il18106","Lur1ngal106","Lurmalb106","Lurkim1106","Lurlfabp106",
                      "Lurcysc106","LbldIL10106","LbldIL6106","LbldPROBNP106","LbldTNI106","LbldTNTHS106",
                      "LbldCKMB106","LbldFABP106","LbldMCP1106","LbldEGF106","LbldVEGF106","delta_scr",
                      "avg_scr")],bootdat$susmild,glm.family="binomial",occam.window=T,
                      prior.param=c(1,rep(0.5,23)))

           ## Choose the model with the highest posterior prob – similar to what was done above
           BMAmodM1<-BMAmod$mle[1,]
           BMA_M1varsBOOT<-BMAmodM1[BMAmodM1 != 0]
           tormM1BOOT<-pmatch(names(BMA_M1varsBOOT),c("(Intercept)","centerFAC."),dup=TRUE,nomatch=0)
           BMA_M1varsNOCBOOT<-BMA_M1varsBOOT[tormM1BOOT==0]

           ## Choose the median prob combination – similar to what was done above
           BMAmodM2<-BMAmod$probne0
           BMA_M2varsBOOT<-BMAmodM2[BMAmodM2 > 50]
           tormM2BOOT<-pmatch(names(BMA_M2varsBOOT),"centerFAC",dup=TRUE,nomatch=0)
           BMA_M2varsNOCBOOT<-BMA_M2varsBOOT[tormM2BOOT==0]

           #### Get apparent (center-adj) AUC (apply models to bootdat)
           ## procedure is VERY similar to getting the apparent center-adjusted AUC in the original data
           ## (only difference is the dataset)

           bootCC<-bootdat[complete.cases(bootdat[,c("centerFAC","cpbtime","Lsercre106","Lbldbnp106",
                      "Lbl1tngal106","Lurcre106","Lur1il18106","Lur1ngal106","Lurmalb106","Lurkim1106",
                      "Lurlfabp106","Lurcysc106","LbldIL10106","LbldIL6106","LbldPROBNP106","LbldTNI106",
                      "LbldTNTHS106","LbldCKMB106","LbldFABP106","LbldMCP1106","LbldEGF106","LbldVEGF106",
                      "delta_scr","avg_scr")]),]

           BMA_M1glmBOOT<-glm(as.formula(paste("susmild",'~',paste(names(BMA_M1varsNOCBOOT),collapse=" +
"),
                              '+ factor(centerFAC)')),data=bootCC,family="binomial", x=TRUE,y=TRUE)
           BMA_M2glmBOOT<-glm(as.formula(paste("susmild",'~',paste(names(BMA_M2varsNOCBOOT),collapse=" +
"),
                              '+ factor(centerFAC)')),data=bootCC,family="binomial", x=TRUE,y=TRUE)

           ## Get linear predictor & AUC for each model
           betaM1BOOT<-BMA_M1glmBOOT$coefficients[2:(1+length(BMA_M1varsNOCBOOT))]
           xbM1BOOT<-as.matrix(bootCC[,names(betaM1BOOT)]) %*% betaM1BOOT
           resultsmatM1BOOT<-as.data.frame(cbind(xbM1BOOT,bootCC$susmild,bootCC$centerFAC))
           allsomersM1BOOT<-sapply(split(resultsmatM1BOOT, resultsmatM1BOOT[,3]),function(x)
                                      somers2(x[,1],x[,2]))[1,]
```

```
	neventsM1BOOT<-sapply(split(resultsmatM1BOOT, resultsmatM1BOOT[,3]),function(x)
sum(x[,2],na.rm=T))
	APPaucM1BOOT<-(allsomersM1BOOT[which(is.na(allsomersM1BOOT)==FALSE)] %*%
				neventsM1BOOT[neventsM1BOOT>0])/sum(neventsM1BOOT,na.rm=T)

	betaM2BOOT<-BMA_M2glmBOOT$coefficients[2:(1+length(BMA_M2varsNOCBOOT))]
	xbM2BOOT<-as.matrix(bootCC[,names(betaM2BOOT)]) %*% betaM2BOOT
	resultsmatM2BOOT<-as.data.frame(cbind(xbM2BOOT,bootCC$susmild,bootCC$centerFAC))
	allsomersM2BOOT<-sapply(split(resultsmatM2BOOT, resultsmatM2BOOT[,3]),function(x)
				somers2(x[,1],x[,2]))[1,]
	neventsM2BOOT<-sapply(split(resultsmatM2BOOT, resultsmatM2BOOT[,3]),function(x)
sum(x[,2],na.rm=T))
	APPaucM2BOOT<-(allsomersM2BOOT[which(is.na(allsomersM2BOOT)==FALSE)] %*%
				neventsM2BOOT[neventsM2BOOT>0])/sum(neventsM2BOOT,na.rm=T)

	## Now, get at the idea of optimism by applying BMA combinations developed in the bootstrap
sample
	## to the original data (nonmissing on the variables in the combination)
	## & calculate the center-adjusted AUC

	BMA1CC<-datfinal[complete.cases(datfinal[,c("centerFAC",names(betaM1BOOT))]),]
		## get a complete-case version of the original dataset – only keep people non-
missing
		## on the variables included in the combination betaM1BOOT
	xbM1appl<-as.matrix(BMA1CC[,names(betaM1BOOT)]) %*% betaM1BOOT
		## calculate linear predictor using the combination estimated in the bootstrap
sample
		## and the complete observations from the original dataset
	resultsmatM1appl<-as.data.frame(cbind(xbM1appl,BMA1CC$susmild,BMA1CC$centerFAC))
		## create new data frame with the linear predictor, the outcome and center
	allsomersM1appl<-sapply(split(resultsmatM1appl, resultsmatM1appl[,3]),function(x)
		somers2(x[,1],x[,2]))[1,]
		## split the data by center and calculate the center-specific AUCs based on the
		## BMA combination developed in the bootstrap sample and the outcome
	neventsM1appl<-sapply(split(resultsmatM1appl, resultsmatM1appl[,3]),function(x)
sum(x[,2],na.rm=T))
		## split the data by center and calculate the number of cases per center
	APPaucM1appl<-(allsomersM1appl[which(is.na(allsomersM1appl)==FALSE)] %*%
		neventsM1appl[neventsM1appl>0])/sum(neventsM1appl,na.rm=T)
		## calculate the center-adjusted AUC as the weighted average of center-specific AUCs

	## same idea as above, but with the median probability combination
	BMA2CC<-datfinal[complete.cases(datfinal[,c("centerFAC",names(betaM2BOOT))]),]
	xbM2appl<-as.matrix(BMA2CC[,names(betaM2BOOT)]) %*% betaM2BOOT
	resultsmatM2appl<-as.data.frame(cbind(xbM2appl,BMA2CC$susmild,BMA2CC$centerFAC))
	allsomersM2appl<-sapply(split(resultsmatM2appl, resultsmatM2appl[,3]),function(x)
		somers2(x[,1],x[,2]))[1,]
	neventsM2appl<-sapply(split(resultsmatM2appl, resultsmatM2appl[,3]),function(x)
sum(x[,2],na.rm=T))
	APPaucM2appl<-(allsomersM2appl[which(is.na(allsomersM2appl)==FALSE)] %*%
		neventsM2appl[neventsM2appl>0])/sum(neventsM2appl,na.rm=T)

	# 6. subtract to get optimism
	optimismM1<-2*(APPaucM1BOOT-0.5)-2*(APPaucM1appl-0.5)
			## estimate the optimism for the maximum posterior prob combination
			## on the Somers’ D scale
	optimismM2<-2*(APPaucM2BOOT-0.5)-2*(APPaucM2appl-0.5)
			## estimate the optimism for the median prob combination
			## on the Somers’ D scale

	c(optimismM1,optimismM2,APPaucM1BOOT,APPaucM2BOOT)
		## output the optimism for the two combinations for optimism-correction
		## output the apparent AUCs in the bootstrap sample – will use these to estimate the
		##	95% CIs for the apparent center-adjusted AUCs
}

## a function to save the warnings in a separate text file
saveAllWarnings <- function(expr, logFile="warning_log.R") {
    withCallingHandlers(expr,
```

```
        warning=function(w) {
            cat(conditionMessage(w), "\n\n",  file=logFile, append=TRUE)
            invokeRestart("muffleWarning")
        })
}

set.seed(10908542)
results<-saveAllWarnings(replicate(1000,bootBMA(1)), logFile="…/warnings1.txt")
           ## run BMA bootstrapping function 1000 times, and give a name to the warning log

rowMeans(results) ## mean optimism and mean apparent center-adjusted AUCs across the bootstrap samples

adjD1<-2*(aucM1-0.5)-rowMeans(results)[1]
           ## (apparent center-adjusted AUC in the original data) – (the estimated mean optimism)
           ## on the somers’ D scale
adjAUC1<-(adjD1/2) + 0.5 ## transform back to AUC scale
adjAUC1 ## optimism-corrected, center-adjusted AUC for the max posterior prob model

## same as above, but for the median prob combination model
adjD2<-2*(aucM2-0.5)-rowMeans(results)[2]
adjAUC2<-(adjD2/2) + 0.5
adjAUC2

## printing the apparent center-adjusted AUCs in the original data
aucM1
aucM2

write.table(results,file="…/BMAresults.csv") ## save all the results in a csv file for later use

apply(results[c(3:4),],1,function(x) quantile(x,probs=c(0.025,0.975)))
           ## gives the 95% CIs based on the quantile bootstrap (2.5th and 97.5th percentiles of the
           ## apparent center-adjusted AUCs across bootstrap samples)
           ## to get 95% CIs for the optimism-corrected, center-adjusted AUC, subtract the estimated
           ## optimism from these intervals (steps given for the max posterior prob combination):
           ## (1) 95% CI for the apparent-center adjusted AUC based on bootstrap quantiles = (LL, UL)
           ##     (given by the “apply” function above)
           ## (2) put on the somers’ D scale: 2*(LL-0.5), 2*(UL-0.5)
           ## (3) subtract off the average optimism: LLnew = 2*(LL-0.5) – rowMeans(results[1]),
           ##                                            ULnew = 2*(UL-0.5) – rowMeans(results[1])
           ## (4) then transform back to AUC scale: (LLnew/2) + 0.5, (ULnew/2) + 0.5
```

Figure S1: Distribution of biomarker combinations in the largest center, stratified by sustained mild AKI case status (scaled).

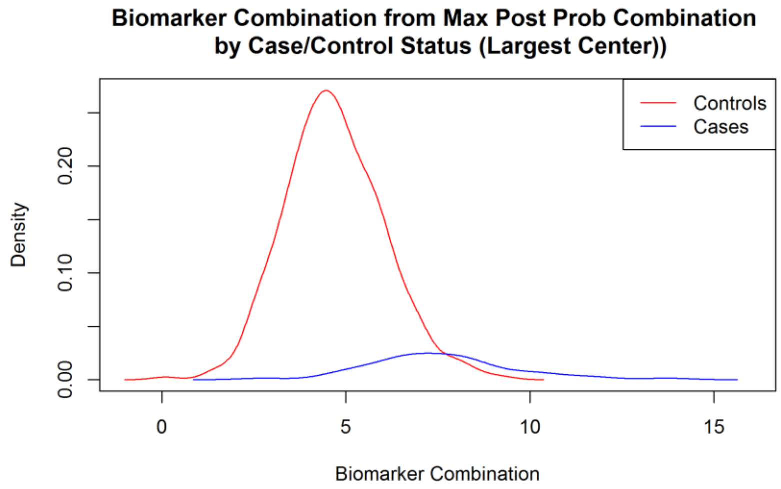

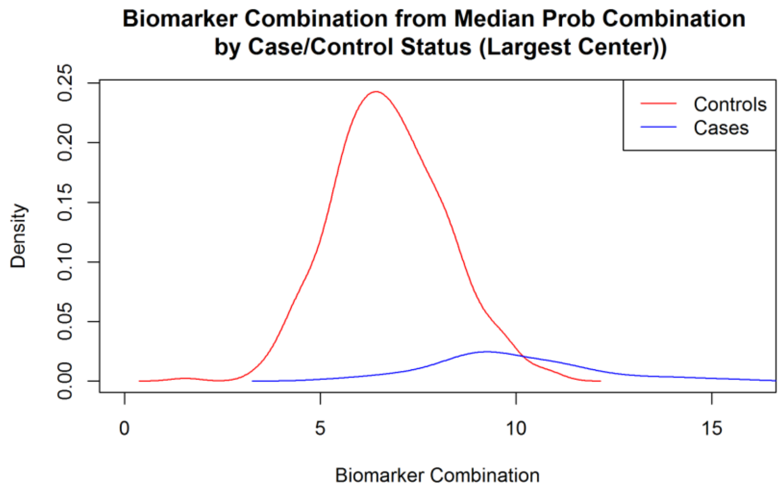

The densities are scaled by the proportion of cases and controls to reflect the prevalence of sustained mild AKI. Abbreviations: AKI = acute kidney injury; max post prob combination = maximum posterior probability combination; median prob combination = median probability combination

Figure S2: Distribution of biomarker combinations in the largest center, stratified by sustained mild AKI case status.

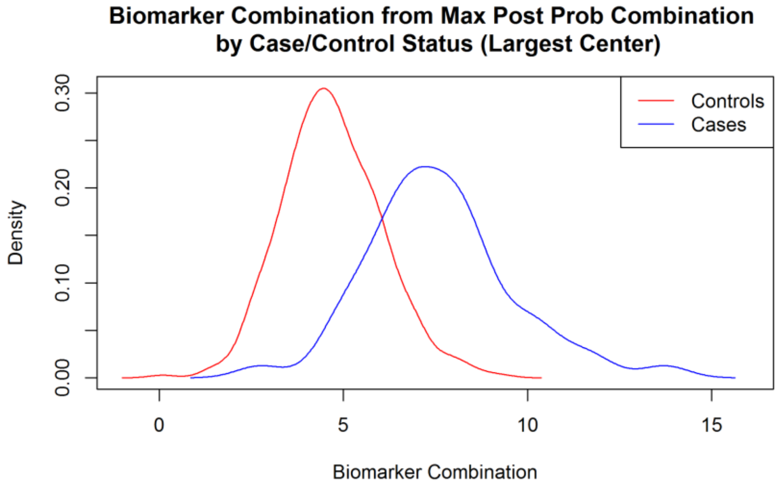

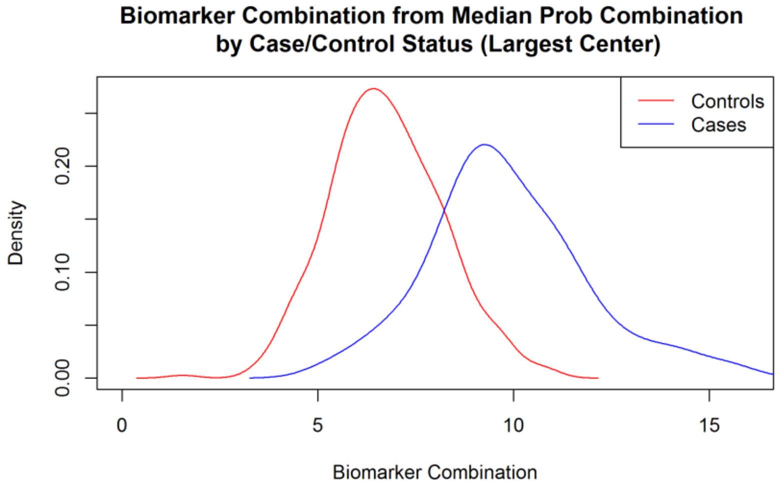

In contrast to Figure S1, the distributions are not scaled to reflect the prevalence of mild AKI cases.
Abbreviations: AKI = acute kidney injury; max post prob combination = maximum posterior probability combination; median prob combination = median probability combination

Figure S3: Distribution of three biomarkers (log plasma NT-proBNP, change in sCr, and log plasma h-FABP) among controls (individuals without sustained mild AKI), stratified by center.

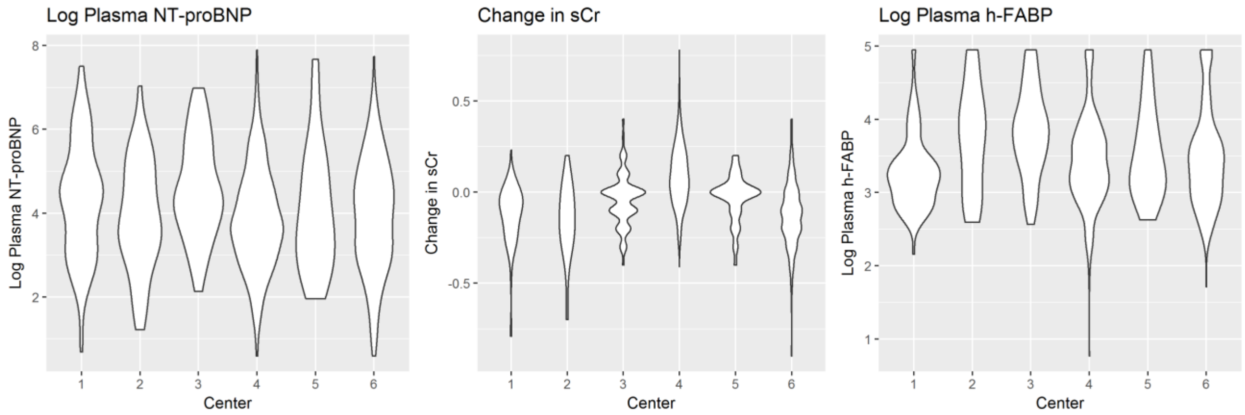

Abbreviations: AKI = acute kidney injury; NT-proBNP = N-terminal-pro-B-type natriuretic peptide, sCr = serum creatinine, h-FABP = heart-type fatty acid binding protein.

Table S1: Posterior variable probabilities for each candidate predictor.

**Predictor**	**Posterior Variable Probability**
Plasma NT-proBNP	100
Change in serum creatinine	100
Plasma h-FABP	75.6
Plasma IL-6	56.5
Urine albumin	8.9
Plasma IL-10	6.3
Plasma EGF	5.7
Plasma TNTHS	4.9
Average serum creatinine	4.5
Urine IL-18	4.1
CPB time	3.6
Serum creatinine	3.4
Urine KIM-1	2.3
Plasma CKMB	1.4
Plasma NGAL	1.3
Urine cystatin C	1.2
Plasma MCP-1	1.1
Plasma BNP	1.0
Urine creatinine	0
Urine NGAL	0
Urine L-FABP	0
Plasma TNI	0
Plasma VEGF	0

Figure S4: Posterior model probability of the combinations selected by the BMA methods across the 1000 bootstrap samples. The first plot corresponds to the maximum posterior probability combination and the second plot corresponds to the median probability combination. “Truncated” means the combination was not considered by the BMA algorithm in that particular bootstrap sample; the truncated value is the minimum posterior model probability in that sample. “Index” indicates the bootstrap sample number.

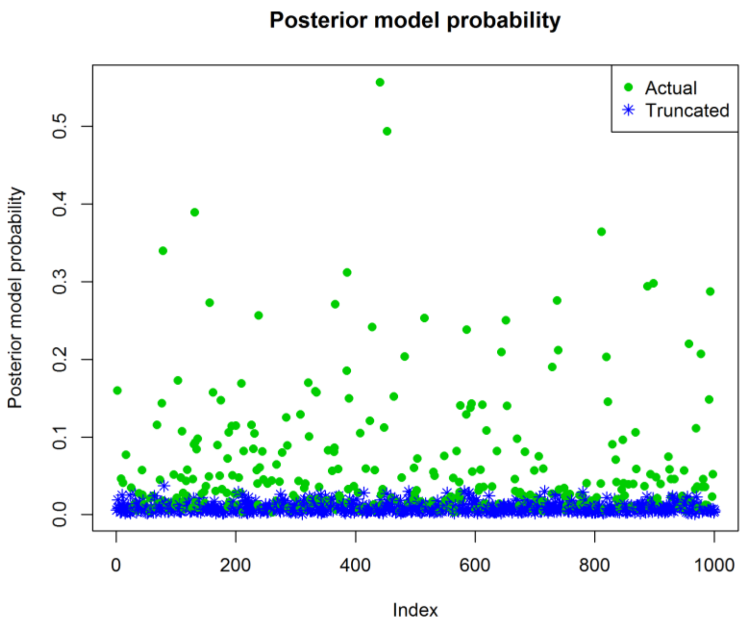

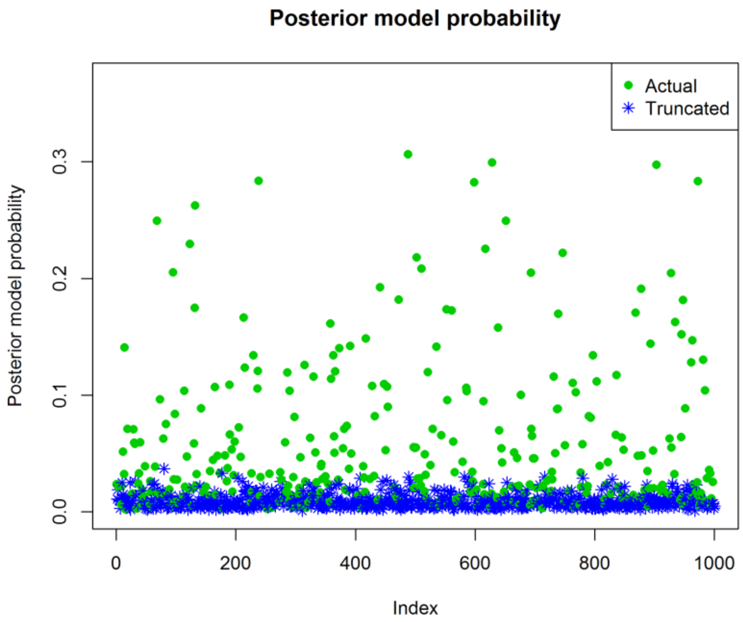

Figure S5: Posterior variable probabilities for each of the candidate predictors across 1000 bootstrap samples. “Index” indicates the bootstrap sample number.

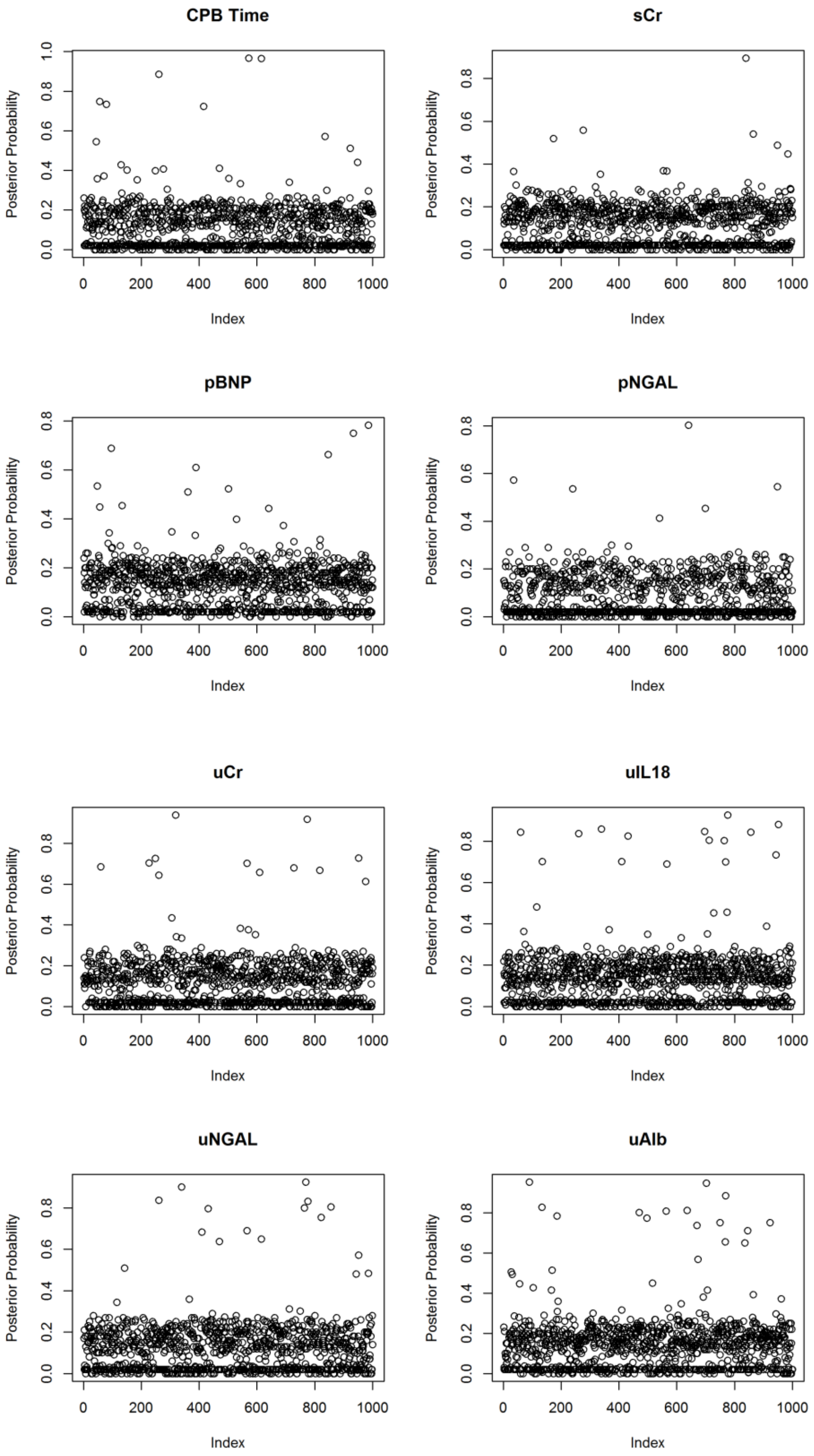

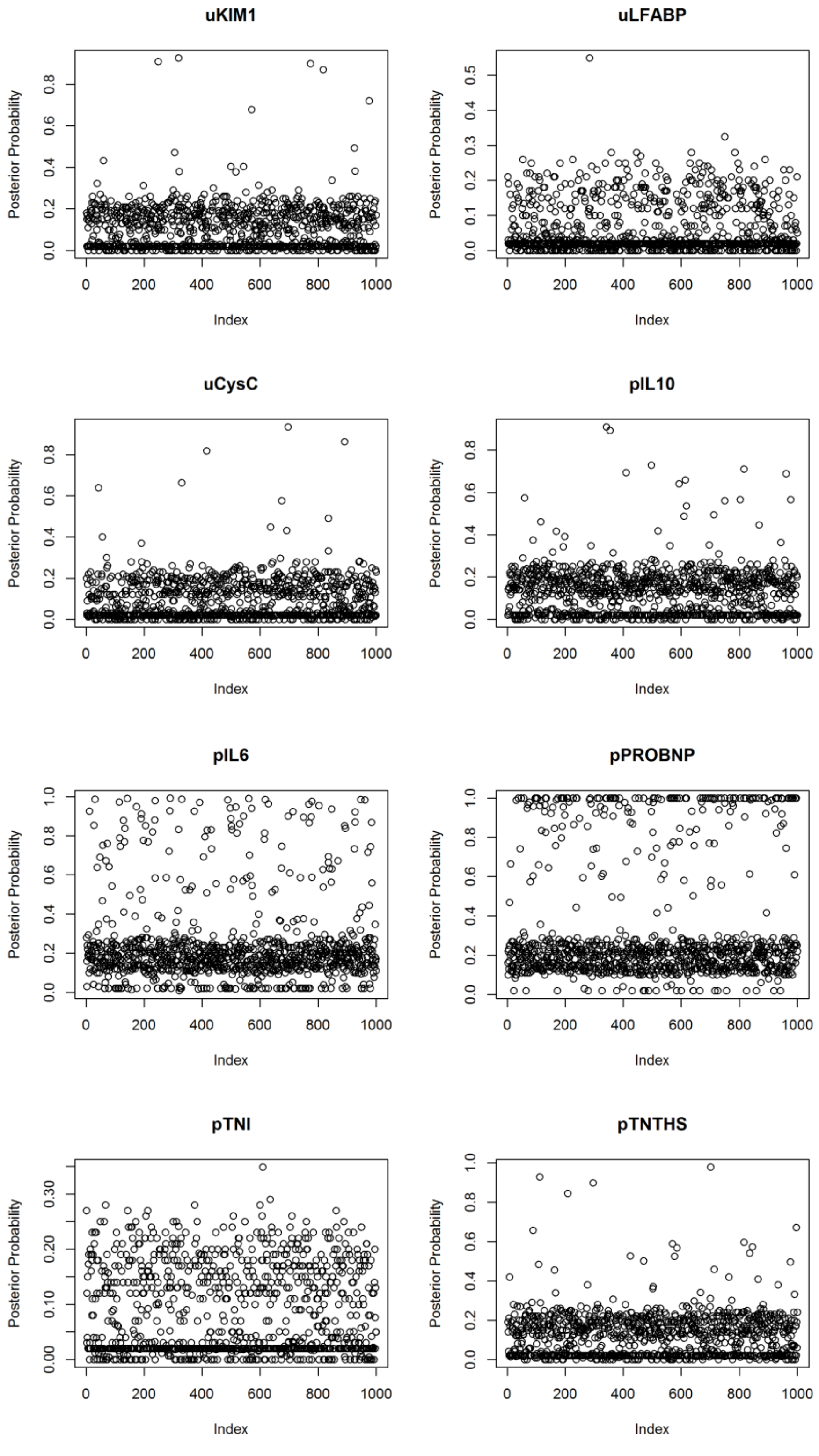
uKIM1
uLFABP
uCysC
pIL10
pIL6
pPROBNP
pTNI
pTNTHS
Posterior Probability
Index
0 200 400 600 800 1000

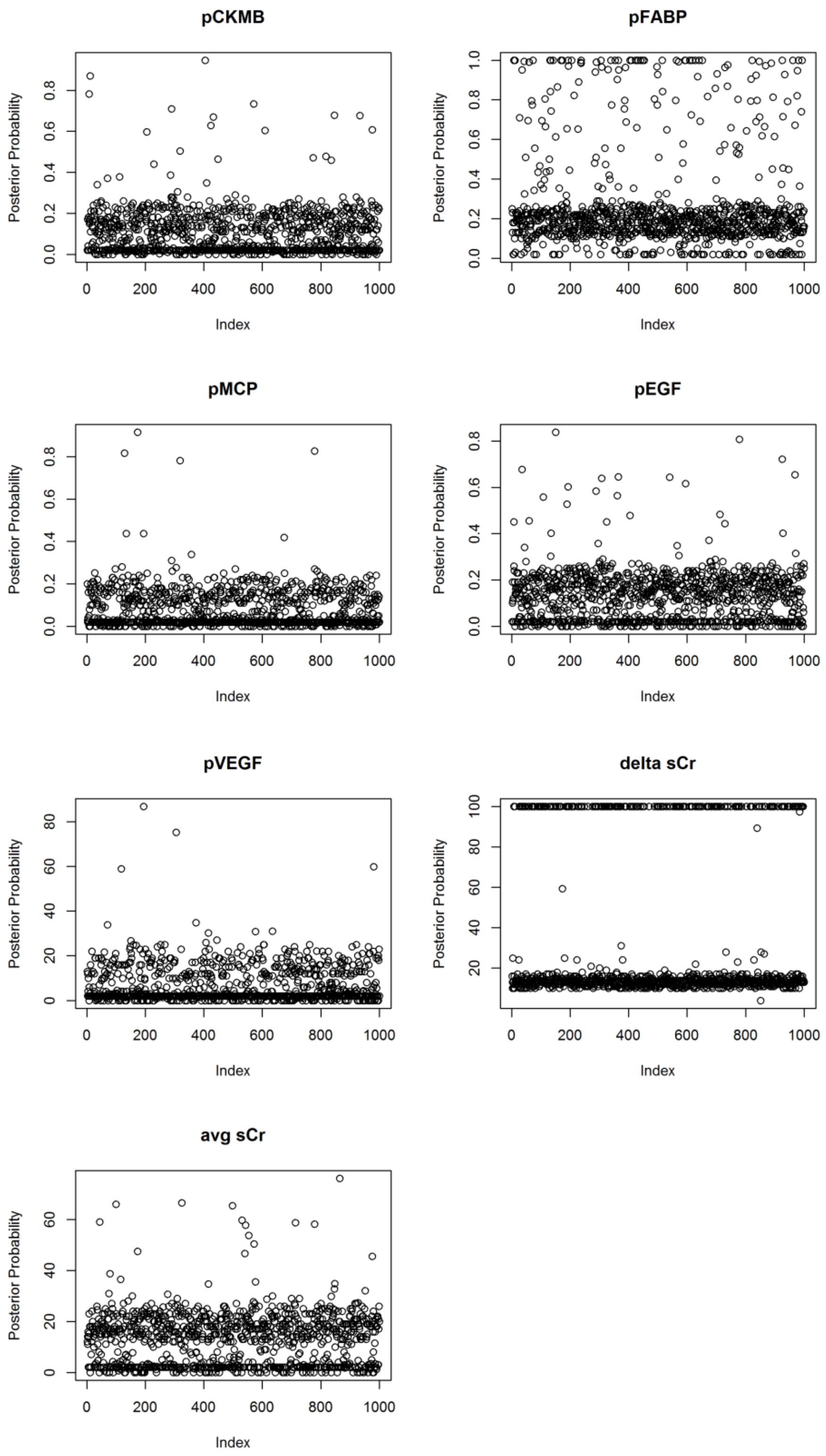
pCKMB
pFABP
pMCP
pEGF
pVEGF
delta sCr
avg sCr
Posterior Probability
Index
0
200
400
600
800
1000

Figure S6: Posterior variable probabilities for each of the candidate predictors when each patient was left out in turn (only observations non-missing on all candidate predictors were included). “Index” indicates the (arbitrary) rank order of the patient in the analysis dataset.

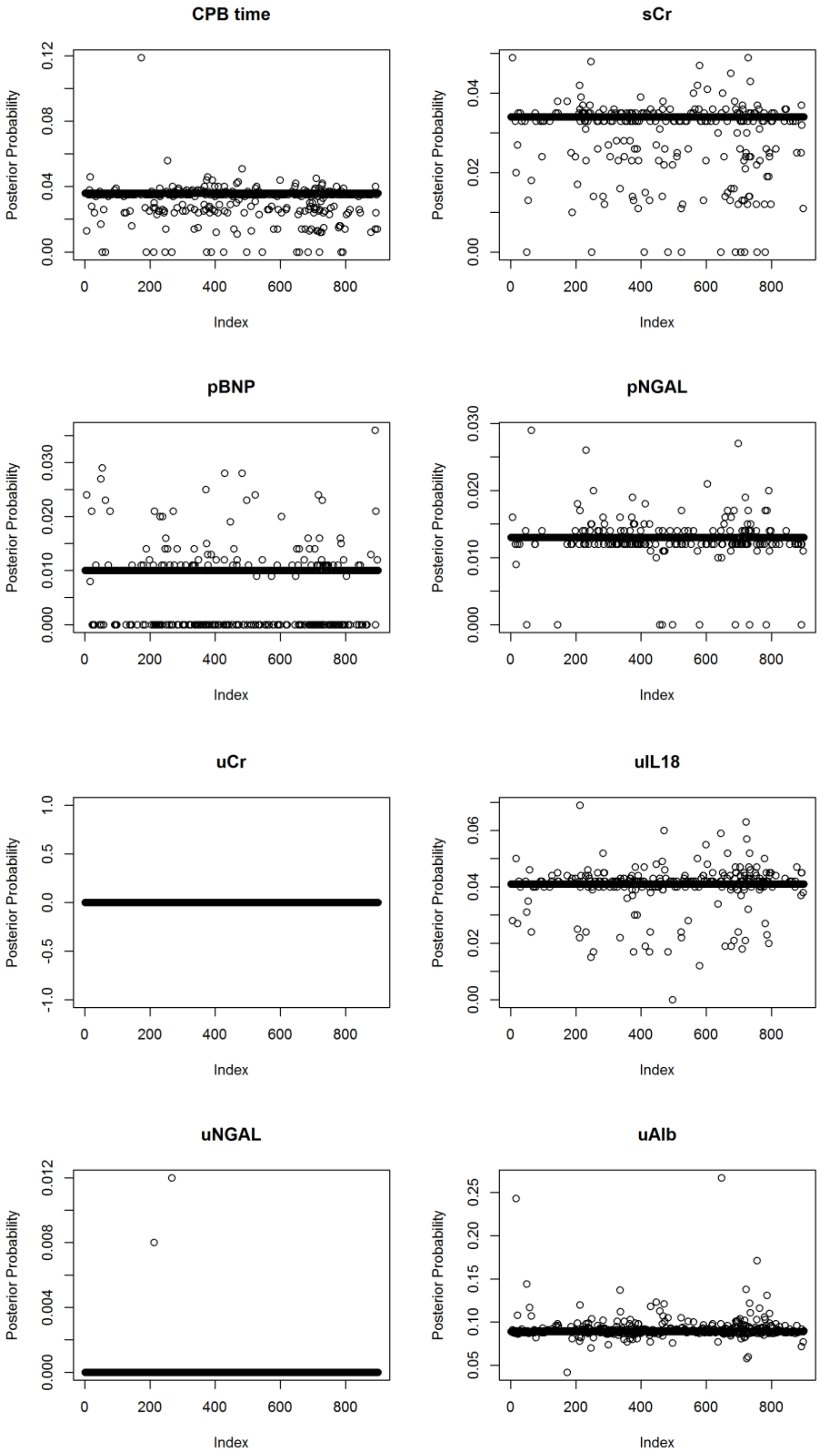

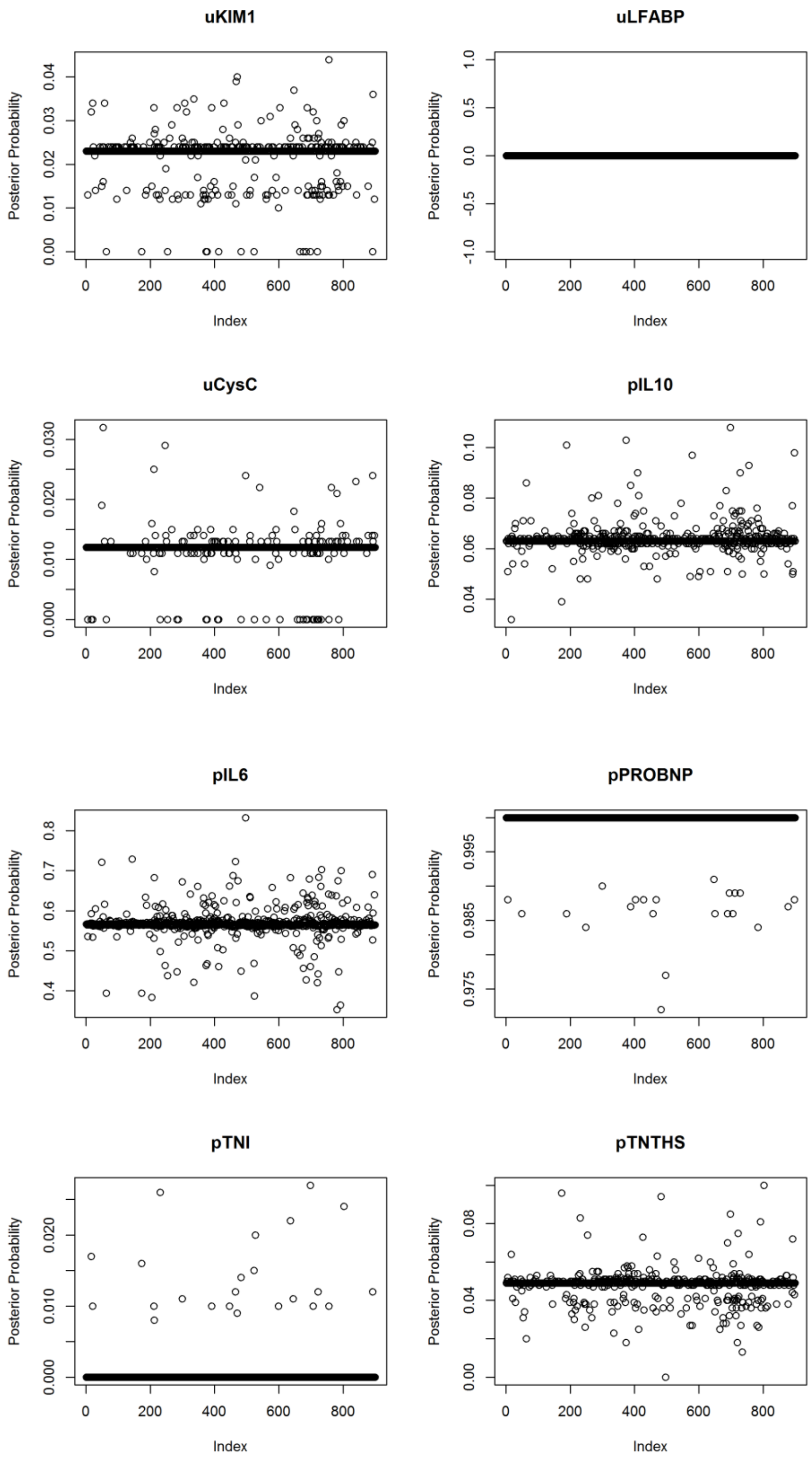
uKIM1
Posterior Probability
0.00 0.01 0.02 0.03 0.04
0 200 400 600 800
Index
uLFABP
Posterior Probability
-1.0 -0.5 0.0 0.5 1.0
0 200 400 600 800
Index
uCysC
Posterior Probability
0.000 0.010 0.020 0.030
0 200 400 600 800
Index
pIL10
Posterior Probability
0.04 0.06 0.08 0.10
0 200 400 600 800
Index
pIL6
Posterior Probability
0.4 0.5 0.6 0.7 0.8
0 200 400 600 800
Index
pPROBNP
Posterior Probability
0.975 0.985 0.995
0 200 400 600 800
Index
pTNI
Posterior Probability
0.000 0.010 0.020
0 200 400 600 800
Index
pTNTHS
Posterior Probability
0.00 0.04 0.08
0 200 400 600 800
Index

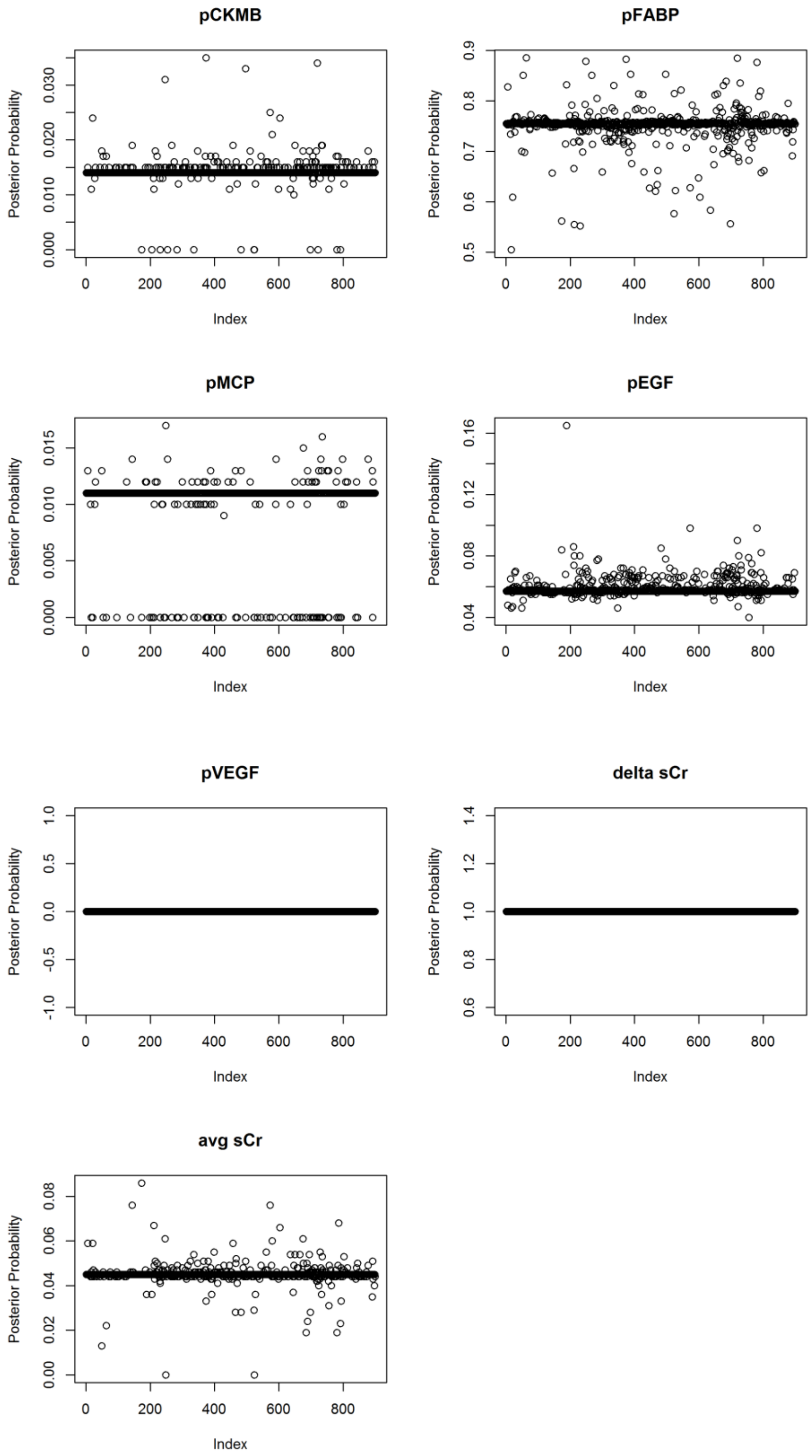
pCKMB
pFABP
pMCP
pEGF
pVEGF
delta sCr
avg sCr
Posterior Probability
Index

Figure S7: Performance (in terms of the center-adjusted AUC) of the estimated selected combinations across 1000 bootstrap samples. The first plot corresponds to the AUC for the outcome of sustained mild AKI; the second plot corresponds to the AUC for the outcome of severe AKI. “Index” indicates the bootstrap sample number.

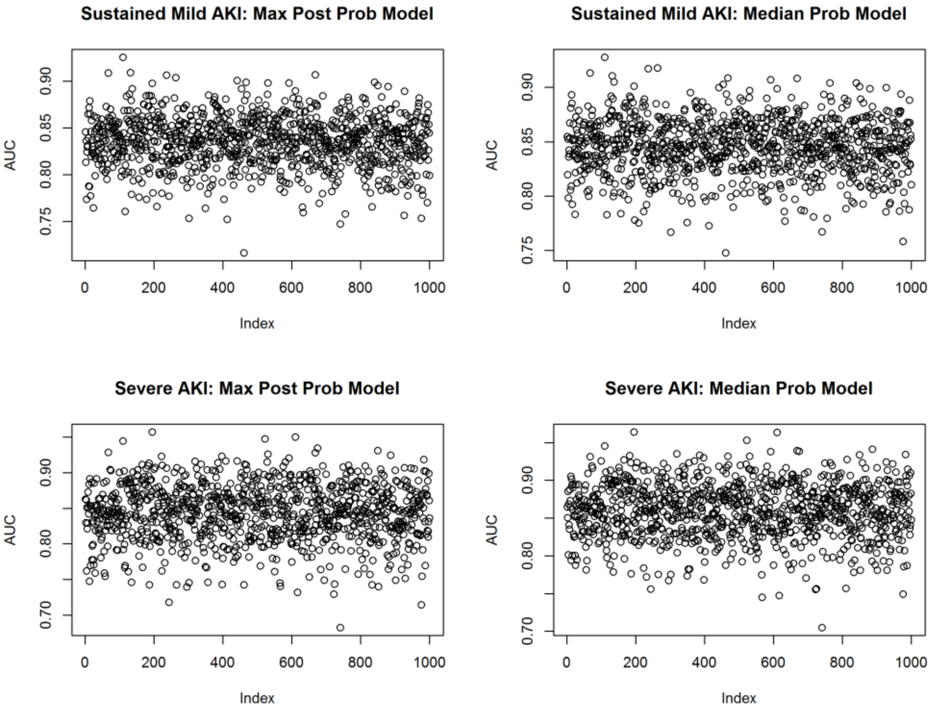

Item S2: Multiple imputation analysis.

In order to explore the impact of deleting observations with missing data, we compared the results of a multiple imputation analysis to the results of our complete-case analysis. First, we created ten completed datasets via multiple imputation using the R package `mice`. Then, we compared results across these datasets.

1. Average of non-zero MLEs across maximum posterior model probability combinations: In each completed dataset, we applied BMA and stored the estimates for the maximum posterior model probability combinations. Then, we averaged the estimates across the ten datasets. Below are the results for variables whose average estimate was not zero: we present the number of imputed datasets where the variable was included in the maximum posterior model probability combination and the average estimated odds ratio (averaged on the log scale) across the ten datasets. We also present the complete-case odds ratio estimates for the variables in the complete-case maximum posterior model probability combination (plasma NT-proBNP, plasma h-FABP, and change in serum creatinine; also provided in Table 3 of the paper) for comparison.

Variable	# Datasets	Average imputation odds ratio	Complete-case odds ratio
**Log plasma MCP-1**	1	0.94	
**Log plasma IL-10**	1	0.98	
**Log plasma IL-6**	9	1.59	
**Log plasma NT-proBNP**	10	1.63	1.60
**Log plasma h-FABP**	10	1.74	2.00
**Change in serum creatinine**	10	1.68 (per 0.1 mg/dL)	1.80 (per 0.1 mg/dL)

Comparing the imputation results to those based on the complete-case analysis, we see that the variables in the maximum posterior model probability combination in the complete-case analysis are in the maximum posterior model probability combination in all ten of the imputed datasets. Plasma IL-6, which was not in the maximum posterior model probability combination in the complete-case analysis, but was in the median probability combination in the complete-case analysis, was in the maximum posterior model probability combination in nine out of ten imputed datasets. In addition, the average odds ratio for these variables is similar to those from the complete-case analysis.

2. We considered two different approaches to summarizing the posterior variable probabilities across imputed datasets.
    a. Average of posterior variable probabilities across imputations: the table below provides the average posterior variable probabilities for the variables whose posterior variable probability averaged across the ten datasets was above 50%. We also present the posterior variable probability estimates from the complete-case analysis (also provided in Table 3 of the paper) for comparison.

Variable	Posterior Variable Probability (Imputation)	Posterior Variable Probability (Complete-case)
**Log plasma IL-6**	0.83	0.57
**Log plasma NT-proBNP**	1.00	1.00
**Log plasma h-FABP**	0.77	0.76
**Change in serum creatinine**	1.00	1.00

Comparing the imputation results to those based on the complete-case analysis, we see that the same variables chosen to be in the median probability combination in the complete-case analysis had an average posterior variable probability above 50% across the imputed datasets. Furthermore, the average posterior variability probability of these variables is similar to that estimated in the complete-case analysis, though the average posterior variable probability for plasma IL-6 is somewhat higher in the imputation analysis.

b. Below we report the number of imputed datasets where the variable's posterior variable probability was above 50% (for variables who achieved this cutoff in at least one dataset).

Variable	# Datasets
**Log plasma MCP-1**	1
**Log plasma IL-10**	1
**Log plasma IL-6**	9
**Log plasma NT-proBNP**	10
**Log plasma h-FABP**	10
**Change in sCr**	10

We see that the variables selected to be in the median probability combination in the complete-case analysis (plasma IL-6, plasma NT-proBNP, plasma h-FABP, and change in serum creatinine) had posterior variable probabilities above 50% in nine or more of the imputed datasets.

3. In each imputed dataset, we applied the BMA methods and evaluated the apparent AUC of the selected combinations. We present the average apparent AUC across the ten datasets. For comparison, we also present the apparent AUCs based on the complete-case analysis.

Combination	Outcome	AUC (Complete-case)	AUC (Imputation)
**Maximum posterior model probability**	Sustained mild AKI	0.84	0.83
	Severe AKI	0.85	0.82
**Median probability combination**	Sustained mild AKI	0.85	0.83
	Severe AKI	0.86	0.82

We see that the average apparent AUC across the ten imputed datasets is somewhat lower than the apparent AUC from the complete-case analysis (note that these estimates have not been corrected for optimistic bias and so differ from the main results reported in the paper). However, the differences in AUC are generally modest.
